# Cattle remain immunocompetent during the acute phase of foot-and-mouth disease virus infection

**DOI:** 10.1186/1297-9716-42-108

**Published:** 2011-10-20

**Authors:** Miriam A Windsor, B Veronica Carr, Bartomiej Bankowski, Debi Gibson, Elizabeth Reid, Pip Hamblin, Simon Gubbins, Nicholas Juleff, Bryan Charleston

**Affiliations:** 1 Pirbright Laboratory, Institute for Animal Health, Ash Road, Woking, Surrey, GU24 0NF, UK; 2 Compton laboratory, Institute for Animal Health, Compton, Berkshire, RG20 7NN, UK

## Abstract

Infection of cattle with foot-and-mouth disease virus (FMDV) results in the development of long-term protective antibody responses. In contrast, inactivated antigen vaccines fail to induce long-term protective immunity. Differences between susceptible species have also been observed during infection with FMDV, with cattle often developing persistent infections whilst pigs develop more severe symptoms and excrete higher levels of virus. This study examined the early immune response to FMDV in naïve cattle after in-contact challenge. Cattle exposed to FMDV were found to be viraemic and produced neutralising antibody, consistent with previous reports. In contrast to previous studies in pigs these cattle did not develop leucopenia, and the proliferative responses of peripheral blood mononuclear cells to either mitogen or third party antigen were not suppressed. Low levels of type 1 interferon and IL-10 were detected in the circulation. Taken together, these results suggest that there was no generalised immunosuppression during the acute phase of FMDV infection in cattle.

## Introduction

Foot-and-mouth disease (FMD) is an extremely contagious and economically important disease of livestock. Outbreaks in normally disease-free countries, such as the UK in 2001 [1] and Japan in 2010 [2], have cost billions of dollars in lost revenue. The current vaccines available for use in endemic countries do not confer long-lasting immunity and highly purified vaccine antigen is required to distinguish between vaccinated and infected animals. Understanding the complex relationship between virus and host is vital in designing new vaccines that can be targeted to those areas of the immune system most likely to induce an effective response.

The causative agent, foot-and-mouth disease virus (FMDV), spreads rapidly between animals and is quickly disseminated within the host, presumably in order to avoid the adaptive immune response (for an overview see Golde et al. [3]).

In cattle, the primary sites of infection in aerosol transmission are the nasopharangeal tissues [4], and associated epithelial tissues [5]. Whilst several studies have examined the host response to FMDV in swine [6-10], little is known about the innate or adaptive response to FMDV in cattle. Type 1 (alpha and beta) interferons (IFN) are induced early in the innate immune response and are considered a dominant factor in shaping both innate and adaptive immune responses [11]. Type 1 IFN certainly seems to play a role in FMD pathogenesis in swine, and Chinsamgaram et al. propose that during infection, type 1 IFN production is regulated by the leader protein of FMDV (L^pro^) [12]. However, prophylactic administration of IFN by adenovirus vector prior to challenge, rapidly induces a protective state in swine [13]. Two studies in swine used direct inoculation of FMDV challenge methods to identify a period of lymphopenia approximately 2 to 4 days post challenge that coincided with peak viraemia [7,14]. In addition, in both studies the animals showed suppression of T cell proliferation in response to mitogen from day 1 to day 7 [14] and day 2 to day 5 or 8 depending on the virus used [7]. Lymphopenia had also been correlated with loss of plasmacytoid dendritic cell (PDC) function and inhibition of T cell function [10]. A study in cattle and Indian buffalo has provided limited evidence of a transient lymphopenia immediately after infection [15]. In swine this immune suppression has also been linked with elevated levels of IL-10 in serum [10]. IL-10 is widely acknowledged to contribute to the anti-inflammatory response and to the inhibition of cellular responses via a variety of mechanisms (for a review see [16]). There is also evidence that natural killer (NK) cells may be functionally defective during infection [17].

In cattle, cytotoxic T lymphocytes (CTL) have been shown to play a role in the FMDV immune response during infection and vaccination [18,19] in a cross serotypic manner [20]. Studies carried out on the proliferative response of cattle peripheral blood lymphocytes following vaccination showed a heterotypic reaction, indicating a sharing of T cell epitopes [21]. When Garcia-Valcarcel et al. inoculated an animal with FMDV, little proliferation was seen until a subsequent re-challenge, when a cross serotype response was observed [22].

The humoral response to FMDV is well documented, with a rapid IgM response switching to IgG [23,24] which confers protective immunity for many years [25]. It has been suggested that this long-lasting antibody response is in part due to the presence of viral particles held by follicular dendritic cells in the lymph nodes of cattle, long after the disease has been resolved [26]. Depletion of T cell subsets by monoclonal antibodies showed that the early antibody response to infection is T cell independent [23].

The aim of the current study was to define the early innate and adaptive immune responses of cattle infected with O serotype FMDV, after they were held in close contact with cattle infected by intra-dermolingual challenge. Specifically, we determined whether there was generalised immune-suppression during the acute phase of FMDV infection in cattle by monitoring the number of leucocytes in the blood and assaying for inflammatory and anti-inflammatory cytokines and suppression of the T cell response. We also determined how rapidly FMDV-specific humoral and cell-mediated immune responses developed.

## Materials and methods

### Infection with FMDV

Male Holstein/Friesian cattle weighing approximately 150 kg were used for these studies. Two animals were exposed to two separate intra-dermolingually challenged cattle (1 × 10^5.7 ^TCID_50 _of cattle-adapted FMDV O UKG 34/2001) for 24 h. These inoculates formed no part of the subsequent study and only the "naturally" exposed animals will be referred to from here on. This procedure was repeated in three sequential studies and the data presented here are an accumulation of these replicates (replicate one = animal C1, replicate two = animals C2 and C3 and replicate three = C4 to C6).

### Vaccination

Five Holstein/Friesian cattle (C7 to C11) were vaccinated intramuscularly with 2 mL of O1Manisa vaccine (O1 Manisa vaccine from the UK FMDV emergency vaccine bank).

### Haematology

Blood samples were collected into anti-coagulant, EDTA, and total leucocyte counts performed on the same day as collection. Each sample was counted in triplicate using a Sysmex haematology analyser (Sysmex F-800 Sysmex corporation Kobe Japan).

### Virus detection

Nucleic acid extraction and analysis was performed using quantitative real-time reverse transcription polymerase chain reaction (qRT-PCR) as described previously [27].

### Virus-neutralising antibody test

Serum samples were tested for anti-FMDV neutralising antibodies as described in the Office International des Epizooties Manual of Diagnostic Tests and Vaccines for Terrestrial Animals. Sera with titres greater than or equal to 45 were considered to be positive [28].

### Liquid-phase blocking ELISA

The liquid-phase blocking ELISA was performed as described by Hamblin et al. [29]. Briefly; virus was bound to immunosorbent plates by being trapped with rabbit anti-O1 Manisa antibody. Test samples of bovine sera were mixed with known standards of guinea pig sera and the resultant competition determined by measuring the amount of guinea pig serum bound to the antigen.

### Interferon assay

Type 1 IFN biological activity was measured in serum samples using an *Mx*/chloramphenicol acetyltransferase (Mx/CAT) promoter-reporter gene assay [30].

### IL-10 ELISA

IL-10 was measured in serum following the method of Kwong et al. [31]. Briefly, ELISA plates were coated with anti-IL-10, cattle sera were applied in duplicate, along with an IL-10 standard series, and detected with biotinylated anti-bovine IL-10. This assay was repeated 3 times.

### Vaccination with commercial bovine herpes virus-1 (BHV) vaccine

Approximately one month prior to FMDV challenge, animals C1 to C6 were immunised with Tracherine (Intervet, NL). Cattle were assayed for proliferative response to BHV antigen immediately prior to challenge with FMDV. However, the BHV-specific T cell proliferative response to the vaccine was variable; as a consequence, only animals with T cell proliferative responses consistently higher than 30 000 cpm prior to FMDV infection (C1, C4, C5 and C6) were included in the analysis of whether FMDV infection significantly affected specific recall responses.

### Proliferation assays

Heparinised blood was diluted with phosphate-buffered saline (PBS) (Invitrogen, Paisley, UK) and centrifuged over Histopaque-1077 (Sigma) at 1328 *g*. Peripheral blood mononuclear cells (PBMC) were collected from the interface, the red blood cells were lysed in erythrocyte lysis buffer (155 mM ammonium chloride, 0.1 mM EDTA and 10 mM sodium bicarbonate, pH 7.2) and PBMC were washed three times with cold PBS. PBMC (2 × 10^5 ^per well) in proliferation medium (RPMI 1640 supplemented with 5% BVDV-free FCS, 1% non-essential amino acids (Invitrogen) 1 mM sodium pyruvate (VWR, Leicestershire, UK), 10 μg/mL gentamicin and 50 μM 2-mercaptoethanol) were incubated with a range of antigens; control and test antigens were added to each well. Antigens included; medium alone, pokeweed mitogen (2.5 μg/mL Sigma), inactivated FMDV antigen, mock infected BHK-21 cell lysate and BHV antigen (heat inactivated prior to use). FMDV antigen was kindly provided by Merial Animal Health, (total antigen concentration was known by the vaccine company but undisclosed). Both FMDV and BHV antigens were previously titrated for use in proliferation assays using PBMC from appropriately vaccinated cattle, with the final concentrations used being 1/1000 for FMDV antigen and 1/100 for BHV. The cultures were incubated for 5 days before 0.037 MBq [^3^H] Thymidine (Amersham, Buckinghamshire, UK) was added to each well. After a further overnight incubation cells were harvested onto filter mats and incorporated radioactivity was measured using a 1450 Microbeta counter (Wallac, Finland).

### Statistical analysis

Linear mixed models were used to investigate the relationship between log10 CPM levels, antigen and time. Model selection proceeded by stepwise deletion of non-significant terms (as judged by the Akaike information criterion), starting from a model including antigen, time (as a factor) and an interaction between them; animal was included as a random effect. A linear mixed model was also used to compare IL-10 levels over time, with the model including time (as a factor) as a fixed effect and animal as a random effect.

## Results

### Viraemia and neutralising antibody responses after challenge with FMDV

**Figure 1:**
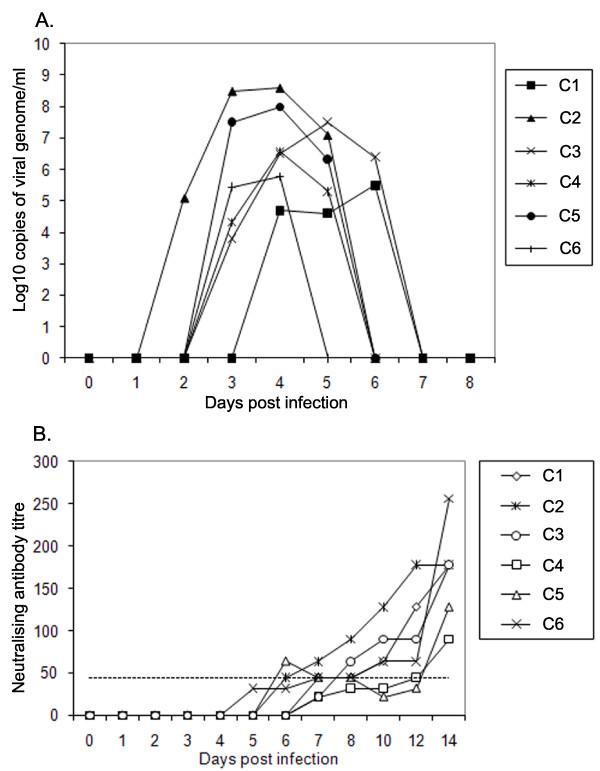
**Cattle infected with FMDV were viraemic during acute infection and developed neutralising antibodies**. **A. **Onset and duration of viraemia of all six FMDV challenged cattle used in this study as obtained by quantitative real-time reverse transcription polymerase chain reaction (qRT-PCR). **B. **Neutralising antibody titre in serum of infected cattle. Sera with titres greater than or equal to 45 were considered to be positive (dotted line).

The onset and duration of viraemia after cattle have been challenged with FMDV has been described previously, and the results obtained in this study are consistent with previous reports [27,32]. In the six animals studied, viraemia was first detected between 2 and 4 days after the animals were placed in contact with FMDV needle challenged animals. Typical clinical signs were observed in all the cattle from 2 to 4 days post infection (data not shown), and viraemia was resolved in all animals by day 7 (Figure 1a). All animals had seroconverted to FMDV as demonstrated by liquid-phase blocking ELISA (data not shown). All these animals had detectable neutralising antibodies seven days after challenge (Figure 1b) and all were considered to have protective titres (> 45) by 14 days after challenge.

### Total circulating leucocyte counts during the acute phase of FMDV infection

**Figure 2:**
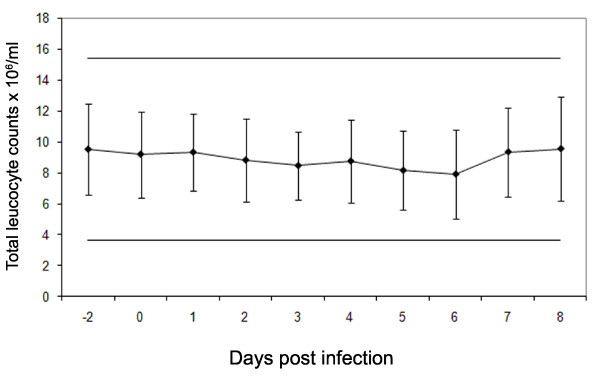
**Total circulating leucocyte counts from all six FMDV challenged cattle (C1 to C6) during the acute phase of FMDV infection**. Blood samples were collected into EDTA, and total leucocyte counts performed on the same day as collection. Counts were performed in triplicate on each sample. (*N *= 6) Lines show 2X STDEV of day 0.

Total circulating leucocyte counts did not significantly fluctuate between day -2 prior to challenge and day 8 after challenge (Figure 2). In addition, the mean circulating leucocyte count of the cattle did not deviate above or below the normal physiological range [33].

Whole lysed blood from two of the cattle (C2 and C3) was also examined for any changes in CD4, CD8, WC1, CD21 and NK cell leucocyte populations [7] but these were not found to vary during the course of infection (data not shown), which is in keeping with the results of Juleff et al. [23].

### Proliferative response of PBMC to mitogen (PWM) during the acute phase of FMDV infection

**Figure 3:**
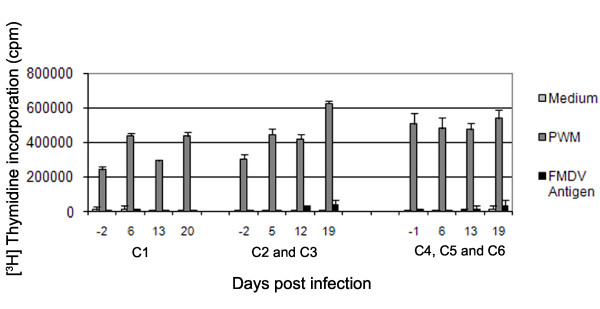
**Proliferative response of PBMC to mitogen during the acute phase of FMDV infection**. PBMC from infected cattle were assayed for proliferation following in vitro incubation with Pokeweed mitogen and FMDV antigen in triplicate. Data shown is the mean of animals in each replicate (C1, C2 and C3 and C4 to C6) for each time point. Error bars show STDEV of all animals in each group for each time point.

Detailed analysis on the proliferative response to Pokeweed mitogen (PWM) in all six animals (C1 to C6) was carried out (Figure 3) (there were minor variations in sample days due to unavoidable differences in sampling schedules between replicates). Marked proliferation, (greater than 200 000 cpm), of PBMC to PWM was detected at various time-points between day -1 prior to FMDV infection and day 19 after challenge.

### Development of FMDV-specific T cell responses during the resolution of acute infection

FMDV-specific T cell responses were measured by proliferation of PBMC to virus antigen in animals C1 to C6 (Figure 3). Although there appears to be a small rise in proliferation to FMDV antigen at day 19 in animals C2 to C6, it was not found to be significant when the data for all animals were exposed to statistical examination. More precisely, there was no significant (*P *= 0.57) interaction between time and antigen, and log10 CPM levels did not change significantly (*P *= 0.31) over time for any of the antigens (medium, FMDV or PWM). There was however, a significant (*P *< 0.001) difference in log10 CPM levels amongst antigens, with levels for PWM being significantly higher than for FMDV or medium (which did not differ significantly (*P *= 0.08) from one another).

### Analysis of established specific memory T cell responses during the acute phase of FMDV infection

**Figure 4:**
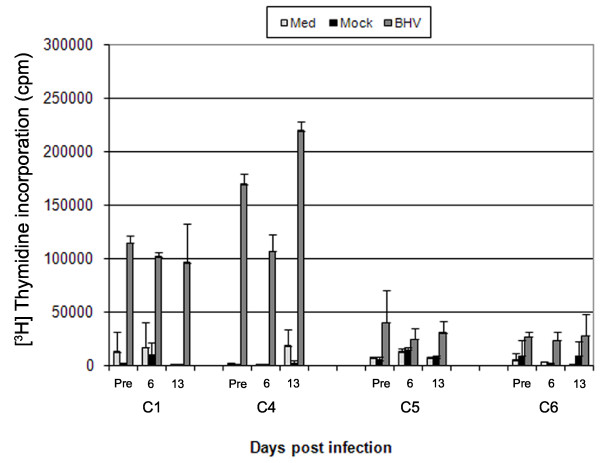
**Proliferative response to third party antigen during the acute phase of FMDV infection**. PBMC from infected cattle (previously vaccinated with BHV) were assayed for proliferation following in vitro incubation with BHV antigen, in triplicate. Due to the variation in magnitude of response to BHV, the data are displayed separately for each of the cattle C1, C4, C5 and C6. Error bars show STDEV of the mean of three wells for each time point.

Vaccination with BHV resulted in variable T cell responses in individual animals (Figure 4), but log10 CPM levels were significantly (*P *< 0.001) higher for BHV compared with medium and mock (log10 CPM levels did not differ significantly (*P *= 0.71) between mock and medium). However, for BHV, there was no significant change in log10 CPM levels at any time point post FMDV infection.

### Development of FMDV-specific T cell responses after vaccination

**Figure 5:**
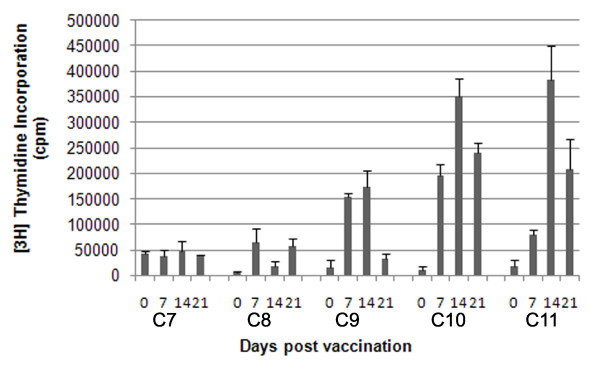
**Proliferative response to FMDV antigen in vaccinated cattle**. PBMC from five cattle vaccinated with FMDV O1 Manisa commercial vaccine were assayed for proliferative response to FMDV antigen. The kinetics of the proliferative response for each animal for the first 21 days post vaccination are shown. Error bars show STDEV for each of the five animals at each time point.

Five cattle (C7 to C11) were vaccinated with O1 Manisa commercial vaccine and examined for T cell responses by PBMC proliferation assays (Figure 5). After day 21 animals C8, C9 and C10 were boosted. An increase in proliferative response was seen in these animals with counts per minute rising to approximately 300 000, 400 000 and 500 000 cpm respectively. The T cell response to FMDV antigen following vaccination was variable in individual cattle, but by inspection, when a response was seen, it was always earlier and of greater magnitude than after infection (it is not appropriate to perform statistical analyses between the studies in Figures 3 and 5).

### Detection of circulating type 1 IFN during the acute phase of FMDV infection

**Figure 6:**
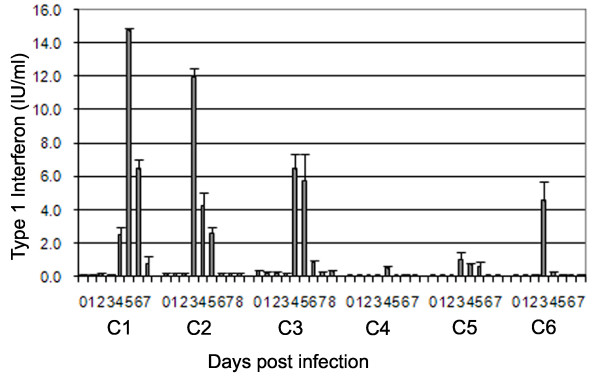
**Type 1 IFN in sera of cattle during the acute phase of FMDV infection**. Six cattle were assayed for circulating biologically active type 1 IFN by Mx CAT reporter assay.

Type 1 IFN was assayed from the sera of six infected cattle (Figure 6). Production of type 1 IFN following infection with FMDV was variable, with the peak values varying between 1 and 14 international units/mL (IU/mL).

### Detection of circulating IL-10 during the acute phase of FMDV infection

**Figure 7:**
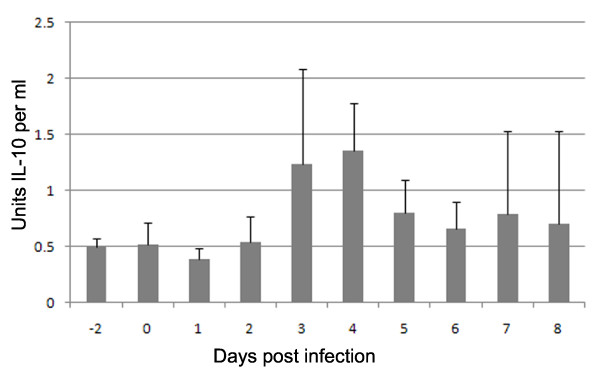
**IL-10 in sera of cattle during the acute phase of FMDV infection**. Six cattle were assayed for circulating IL-10 by ELISA from day -2 to day 8 post infection. Data shown is the mean of all six animals (C1 to C6). Error bars show STDEV of the mean.

Six cattle (C1 to C6), were assayed for circulating IL-10 before, during, and after the acute phase of FMDV infection (Day -2 through to day 8, Figure 7). Low, but statistically significant compared to pre-challenge (*P *< 0.03), levels of IL-10 were detected in all animals, rising over time, reaching a peak on average of 1.35 IU/mL between day 3 and 4, after which they declined.

## Discussion

Early studies by Cunliffe et al. [25] established the long duration of immunity following infection with FMDV in cattle. In contrast, current commercial vaccination protocols require regular re-inoculation to maintain immunity. Analysing the differences in the immune response in the natural host during these two processes may lead to the design of more effective vaccines.

By exposing six cattle to FMDV this study aimed to; investigate whether a generalised suppressive state is induced, compare early T cell responses to those of vaccinates, and examine the expression of key immune-modulatory cytokines.

After natural exposure to FMDV, viraemia is typically detectable within 2 to 3 days followed by clinical signs [27]. Production of high titres of neutralising antibodies can be detected as early as six days following natural infection, by a process shown previously to be T cell independent in cattle [23]. These previous observations were confirmed in all of the cattle used in this study. Compared to the rapid production of antibody, significant FMDV-specific T cell responses (assayed by proliferation of PBMC) were not observed following natural infection. These data are supported by other cattle studies, with no FMDV-specific T cell response detectable up to 32 days post exposure in some cases (M. Windsor unpublished data). We also investigated whether there was any loss of mitogen or specific antigen induced T cell proliferative responses during the course of acute infection. Abrogation of proliferation of PBMC to mitogen in pigs occurs between 2 and 8 days but most noticeably at day 5 after needle challenge [7]. Following natural challenge, peak viraemia in cattle occurs at least two days later than needle challenge [34], which is consistent with the pattern of viraemia in our study. We did not observe any loss of mitogen or specific antigen induced T cell proliferative responses on day 6 post-infection. Even though a specific T cell response to FMDV could not be detected during the acute stages of infection, the T cell proliferative responses to mitogen and a third party antigen (BHV) were unaffected.

In a previous study using bovine dendritic cells (DC), Robinson et al. [35] showed that, in vitro, DC infection was enhanced by the formation of FMDV immune complexes. These data suggest that as FMDV rapidly loses its ability to infect susceptible cells due to increasing neutralising antibody titres, it gains the capacity to infect immune cells via CD32 (FcγR). These findings by Robinson et al. suggest that infection and killing of DC by immune-complexed FMDV may be responsible in part for the delayed FMDV-specific T cell proliferative response [35]. Clearly, it is not the ability of the T cells to respond that is affected, as evidenced by the maintenance of established T cell responses to mitogen and third party antigen.

The FMDV-specific T cell proliferative response in the five vaccinates was variable. Where an effective response to antigen was seen, it was of a high magnitude and detectable from seven days post vaccination. It should be noted that although the initial response was low in some cattle, they all subsequently went on to develop a similar magnitude of T cell response following a booster vaccination, showing that there was no underlying impairment of the response in these animals.

Transient lymphopenia has been observed in swine following infection with FMDV which correlated with the virulence of the isolate used [7]. In cattle, we were unable to detect any evidence of lymphopenia, with the numbers of leucocytes remaining constant and within physiological ranges during the period of viraemia and clinical FMD. Leukocyte subsets were also examined in whole lysed blood in two of the animals and no changes were observed, confirming our previous observations [23]. This contrasts studies in pigs, where a significant loss of circulating CD4^+ ^and CD8^+ ^T cells was observed [7].

Studies in pigs have found high levels of type 1 IFN following experimental infection with several FMDV isolates [10,36], which correlates with immune suppression. Due to the different methods of quantification used in these studies, it is problematic to compare the levels of IFN produced. In cattle we have measured the specific activity of type 1 IFN (in international units), whereas total circulating type 1 IFN protein was measured in pig serum. However, this IFN is not necessarily biologically active. Several sources, including manufacturers' data sheets, give type 1 IFN specific activity (cross species) at between 1 and 3 × 10^8 ^IU/mg of total IFN. Using this calculation we can speculatively compare type 1 IFN in the sera of pigs and cattle during FMDV infection, and postulate that pigs do indeed produce more IFN than the cattle in this study. The most compatible study in pigs was performed by Nfon et al., who used O1Campos challenge [10]. At least 9 fold more type 1 IFN was produced in pigs compared to our results in cattle. In pigs, IFN clearly has an important role to play in the resolution of infection, as the administration of type 1 IFN in a viral vector protects against subsequent challenge with FMDV [37].

Our study implies that cattle produce very little type 1 IFN in comparison to pigs, which is in keeping with previous studies [38], yet cattle still resolve the disease effectively. Depletion of CD4^+ ^cells in cattle during infection further reduces circulating type I IFN, but infection is still resolved [23], indicating that the presence of type 1 IFN in the circulation may not have any bearing on the resolution of disease. The IFN detected in the circulation during FMDV infection in cattle is thought to be produced by CD4^+ ^PDCs interacting with immune-complexed virus [39]. The PDCs found in cattle secondary lymphoid tissue are capable of producing large amounts of IFN in vitro [39]. It is possible therefore, that IFN does play a local role at the sites of infection such as lesions, or in the lymph nodes, and that the IFN found in the circulation is derived from these high concentration sources. Summerfield et al. and Nfon et al. found large numbers of circulating PDCs in pigs [10,40] in comparison to the low numbers detectable in cattle. Nfon also found that these PDCs were functionally impaired during the latter stages of infection with FMDV.

Low levels of IL-10, corresponding with the peak of clinical signs, were found in the serum of the cattle studied. Whilst IL-10 does appear to play a role in immune suppression in pigs during infection with FMDV [8], it is most commonly associated with the maintenance of chronic infections such as Hepatitis C in humans [41,42] and Mycobacterium Bovis in cattle [43]. Studies in mice have shown that FMDV infected DC can stimulate splenic CD9^+ ^B cells to produce T-independent neutralising IgM antibodies, via an IL-10 dependent process [44]. We propose that the absence of leucopenia and immunosuppression in cattle, during acute FMDV infection, is associated with the low levels of type 1 IFN and IL-10. These differences in cytokine profile between pigs and cattle may also explain why, in general, more severe clinical signs are seen in pigs infected with FMDV [45].

## Competing interests

The authors declare that they have no competing interests.

## Authors' contributions

MW carried out the circulating leucocyte counts, proliferation assays of infected cattle, IL10 ELISAs, FACS analysis, analysis and interpretation of data and wrote the manuscript. BVC carried out proliferation assays of vaccinated cattle. BB conducted in vivo experiments, determined viraemia and contributed to experimental design. DG conducted in vivo experiments determined viraemia and carried out competition ELISAs. ER carried out Interferon assays. PH carried out VNT assays. SG carried out statistical analysis. NJ conducted in vivo experiments, carried out IFN assays, coordinated experimental design, analysis and interpretation of data and assisted with manuscript. BC initiated experimental design, analysis and interpretation of data and assisted with manuscript. All authors have read and approved the final manuscript.

## Authors' Information

BC is a Jenner investigator and NJ is a Welcome Trust intermediate clinical fellow.
